# Tertiary lymphoid structures in pulmonary granulomas of cattle experimentally infected with aerosolized *Mycobacterium bovis*

**DOI:** 10.1186/s12917-025-04804-x

**Published:** 2025-06-05

**Authors:** Mitchell V. Palmer, Douglas E. Jones, Nicholas J. Bockenstedt, Paola M. Boggiatto

**Affiliations:** 1 United States Department of Agriculture, Infectious Bacterial Diseases of Livestock Research Unit, National Animal Disease Center, Agricultural Research Service, 1920 Dayton Ave, Ames, IA 50010 USA; 2 Department of Veterinary Pathology, College of Veterinary Medicine, Iowa State University, 1600 S 16 th St., Ames, IA 50011 USA

**Keywords:** Bovine, Granuloma, Tertiary lymphoid structure, Lymph node, Mycobacteria, Tuberculosis

## Abstract

*Mycobacterium bovis* is the primary cause of tuberculosis in animals, most notably cattle. In cattle and other susceptible hosts, the hallmark lesion of tuberculosis is the granuloma. Granulomas represent the host–pathogen interface where disease outcome is determined; therefore, it is critical to understand host–pathogen interactions at the granuloma level. Granulomas are highly structured lesions with distinct cellular compartments for T cells, macrophages, multinucleated giant cells, and B cells. A recognized but poorly understood morphologic feature of many granulomas is the presence of structures resembling follicular or germinal center-like arrangements of B cells known as tertiary lymphoid organs, or tertiary lymphoid structures. Pulmonary granulomas from cattle experimentally infected with *M. bovis* were collected at 15-, 30-, 90-, 180- and 270-days post-infection and examined for the presence of tertiary lymphoid-like structures. Follicle-like structures associated with granulomas were first seen 90 days after infection and persisted in later time points. Compartmentalization of T cells and B cells similar to follicles in germinal centers of lymph nodes was demonstrated using in situ hybridization. Additionally, the presence and arrangement of myeloid cells, endothelial cells, T follicular helper cells, and chemokines critical to tertiary lymphoid structure formation was shown to be similar to lymph node follicles and that described for tertiary lymphoid structures in other species. This represents the first demonstration by in situ hybridization of the similarities of follicle-like structures associated with pulmonary bovine tuberculous granulomas to tertiary lymphoid structures in other species and follicles within secondary lymphoid organs such as lymph nodes.

## Introduction

Bacteria of the genus *Mycobacterium* are Gram-positive, acid-fast bacilli (AFB). Among the 195 mycobacterial species [1], several are important human and animal pathogens such as *Mycobacterium tuberculosis* and *Mycobacterium bovis*. *Mycobacterium bovis* is the primary cause of tuberculosis (TB) in numerous mammals, most notably cattle. It can also cause disease in humans that is indistinguishable from disease caused by *M. tuberculosis*, the more common cause of TB in humans. The hallmark lesion of tuberculosis, regardless of host or tissue, is the granuloma. The granuloma is a morphologically distinctive microscopic lesion dominated by epithelial-like (epithelioid) macrophages, lymphocytes, and multinucleated giant cells. In humans and animals, tuberculous granulomas represent the host–pathogen interface where disease outcome (i.e., dissemination, confinement, or resolution) is determined; therefore, it is critical to understand host–pathogen interactions at the granuloma level [2–4].

In cattle the heterogenous nature of granulomas has been described in terms of morphology, bacterial burden, cellular composition, and cytokine expression [5–13]. Although considerable heterogeneity exists among bovine granulomas, spatial organization is such that central regions of necrosis are surrounded by macrophage-rich zones where many macrophages have undergone the process of epithelization to become epithelioid macrophages [14]. Lymphocyte rich populations are largely peripheral to the macrophage-rich zone and may be found within or external to variable bands of collagen forming an outermost capsule. These lymphocytes may be loosely arranged or organized into follicular or germinal center-like arrangements, similar to that seen in secondary lymphoid organs (SLOs) such as lymph nodes, and variously known as ectopic lymphoid-like structures (ELS), tertiary lymphoid follicles (TLF), tertiary lymphoid organs (TLO), or tertiary lymphoid structures (TLS) [15–19].

The paradox of the granuloma is that this host response is necessary to control infection while also providing a niche for bacillary persistence and can result in excessive host-driven tissue damage [20]. Protective immune responses against tuberculous mycobacteria are associated with cell-mediated immunity, specifically T helper (T_H_1) CD4 + T cells [21, 22]. While CD4 + T cells are essential in control of *M. bovis* infection, the fact that *M. bovis* persists in most infected cattle and that sterile granulomas are uncommon [23], demonstrates that in many cases these responses are insufficient to clear the infection. However, infection with tuberculous mycobacteria has been shown to provide resistance to subsequent infection by the same organism, a phenomenon referred to as premunition or concomitant immunity, the establishment of which is dependent upon a persistent infection and maintenance of an effector CD4 + T cell population [24–27]. It has been suggested that the complex environment of the granuloma shapes and limits effective T cell responses. Specifically, that the peripheral location of most T cells in the granuloma is distant from the bacilli in the macrophage-rich regions preventing direct interaction between CD4 + T cells and infected macrophages [15].

In the early twentieth century, the belief was that host defenses against intracellular pathogens such as *M. bovis* were limited to cell-mediated immune responses and that responses to extracellular pathogens were mediated by antibodies produced from B cells [28]. Importantly, B cells not only produce antibodies with diverse isotypes, some of which support macrophage activation, but they are also competent antigen presenting cells and produce various cytokines that can influence the function of a broad range of immune cells including T cells and macrophages [28]. In tuberculous granulomas of mice, non-human primates, and humans, B cells may form prominent TLSs [29–33].

The term TLS is used to describe the non-ontogenetic formation of lymphoid structures, composed of lymphocytes and stromal elements at non-lymphoid sites, such as the granuloma; therefore, by definition, TLSs arise in tissues whose main purpose is a functionality other than the generation of immune cells or the initiation of an adaptive immune response [34]. These structures may form in response to chronic or persistent stimuli such as inflammation, microbial infection, autoimmune responses, or neoplasia [35]. Their structure and cellular composition can range from simple B and T cell aggregates [17], to highly ordered compartmentalized T and B cell areas with germinal centers [36]. They contain fibrovascular stromal elements including high endothelial venules (HEV), resembling SLOs such as lymph nodes [35]. Tertiary lymphoid structures differ from SLOs in that they are more heterogenous, not limited to a fixed location, non-encapsulated, and develop postnatally. Moreover, development of SLOs is preprogrammed, while initiation of TLSs relies on an inducible trigger such as chronic inflammation [36].

Beyond their lymph node-like nature (i.e., compartmentalized T and B cell areas with a follicle-like arrangement, and fibrovascular stromal elements) TLSs also express homeostatic chemokines such as CXCL13, CCL19 and CCL21, which play an important role in TLS formation and maintenance [18]. Although the presence of follicle-like structures in bovine tuberculous granulomas has been noted in histopathologic descriptions [9–11, 13, 37], the objective of this study was to identify follicle-like structures in bovine pulmonary tuberculous granulomas, examine their temporal development, and determine if these follicle-like structures recapitulate the features of TLSs.

## Methods and materials

### *Mycobacterium bovis* aerosol challenge

*Mycobacterium bovis* strain 10–7428 was used for all experimental infection in this study. This field strain was of low passage (< 3) and had been shown previously to be virulent in the calf aerosol model [38]. Inoculum was prepared using standard techniques [39] in Middlebrook’s 7H9 liquid media (Becton Dickinson, Franklin Lakes, NJ, USA) supplemented with 10% oleic acid-albumin-dextrose citrate (OADC; Difco, Detroit, MI, USA) plus 0.05% Tween 80 (Sigma Chemical Co., St. Louis, MO, USA). Mid log-phase growth bacilli were pelleted by centrifugation at 750 xg, washed twice with phosphate buffered saline (PBS) (0.01 M, pH 7.2) and stored at −80 ^0^C until used. Frozen stock was warmed to room temperature (RT) and diluted to the appropriate cell density in 2 ml of PBS. Bacilli were enumerated by serial dilution plate counting on Middlebrook’s 7H11 selective media (Becton Dickinson). A single dose was determined to be 1.12 X 10^4^ CFU per calf.

Twenty-five Holstein steers, approximately 9 months of age, were obtained from a bovine TB (bTB) free source in a US state determined to be bTB free by the USDA and housed at the National Animal Disease Center (NADC) campus. Prior to challenge, calves were moved into an agricultural biosafety level 3 (AgBSL3) facility and allowed to acclimate for approximately 2 weeks. Aerosol infection of calves with virulent *M. bovis* has been described in detail previously [6, 38, 40]. Briefly, calves were infected with a single dose of virulent *M. bovis* strain 10–7428 by nebulization of inoculum into a mask (Equine AeroMask®, Trudell Medical International, London, ON, Canada) covering the nostrils and mouth. Five non-infected control age-matched calves were housed separately in the same AgBSL3 facility. All experimental animal procedures were conducted in accordance with recommendations in the Care and Use of Laboratory Animals of the National Institutes of Health and the Guide for the Care and Use of Agricultural Animals in Research and Teaching [41, 42]. Procedures were also approved by the USDA-National Animal Disease Center Animal Care and Use Committee.

### Sample collection

Five steers each were euthanized at 15, 30, 90, 180 and 270 days after infection. Steers were humanely euthanized by intravenous administration of sodium pentobarbital. A single non-infected steer was similarly euthanized and examined at each time point. Tissues were examined for gross lesions and processed for microscopic analysis as described previously [38]. More complete descriptions of temporal lesion formation and isolation of *M. bovis* from granulomas in this cohort are found in previous publications [5, 23]. Pulmonary lesions suspected to be tuberculous granulomas were dissected out and processed as individual samples. A maximum of 5 tissue sections (≤ 0.5 cm in width) were collected from each of the five lung lobes for a total of 25 granulomas per steer. Tissue samples were fixed by immersion in 10% neutral buffered formalin (≥ 20 volumes fixative to 1 volume tissue) for approximately 24 h (hrs) and transferred to 70% ethanol, followed by standard paraffin embedding techniques. Paraffin embedded samples were cut in 4 μm thick sections, transferred to Superfrost Plus™ charged microscope slides (Thermo Fisher Scientific, Pittsburg, PA) and stained with hematoxylin and eosin (HE). Numerous near-adjacent unstained sections were used for in situ hybridization (ISH) analysis. Tracheobronchial lymph nodes from age-matched non-infected steers were collected and processed in a similar fashion for comparison to granuloma-associated TLSs.

### Microscopic examination

Using a modification of granuloma staging described by Wangoo et al. in cattle [6, 7, 11] and Rayner et al. in rhesus macaques [43], granulomas in each microscopic section were staged (1 to 5). Type 1 lesions were unorganized, lacking defined boundaries or peripheral lymphocyte-rich zones and were composed of small single or multiple foci of macrophages and few lymphocytes which expanded alveolar septa and extended into the alveoli. Type 2 lesions were composed of similar inflammatory cells as type 1 lesions, still lacking a peripheral lymphocyte-rich zone but were more circumscribed, roughly circular with variably demarcated borders compared to type 1 lesions. Type 3 lesions were similar to type 2 lesions but contained small areas of necrosis, characterized by loss of cellular detail, nuclear pyknosis and karyorrhexis. Type 4 lesions were organized, well circumscribed granulomas consisting primarily of macrophages admixed with lesser numbers of neutrophils and variable numbers of peripheral lymphocytes (non-necrotizing granulomas). Type 5 lesions were similar to type 4 lesions but exhibited central necrotic foci (necrotizing granulomas).

### Messenger RNA (mRNA) chromogenic ISH

A total of 5 granulomas from each of 5 lung lobes from each of 25 steers were examined. RNAscope® ZZ probe technology (Advanced Cell Diagnostics, Newark, CA) was used to perform mRNA ISH in formalin-fixed paraffin-embedded (FFPE) tissue sections using the RNAscope® 2.5 HD Reagents – RED kit (Advanced Cell Diagnostics). Proprietary ZZ probes (Advanced Cell Diagnostics) complementary to mRNA sequences of interest were used for visualization of mRNA transcripts for the following markers: CD79 A (Cat # 1159921-C1); CD3ε (Cat # 1217121-C1); CXCL13; (Cat # 1284931-C1); CD34 (Cat # 1284951-C1), CCL19 (Cat # 1303581-C1), CCL21 (Cat # 1284941-C1), integrin subunit alpha M (ITGAM; CD11b; Cat #1303591-C1), integrin alpha X (ITGAX, CD11c; Cat # 1303601), ICOS (Cat # 1285661). A positive control probe targeted the *Bos taurus*-specific *cyclophilin B* (*PPIB*) housekeeping gene (Cat # 3194510), while a probe targeting *dapB* of *Bacillus subtilis* (Cat # 310043) was used as a negative control. The RNAscope® labelling technique has been shown to be capable of single mRNA molecule detection [44].

Formalin-fixed paraffin-embedded tissue pretreatment was performed manually with antigen retrieval according to the manufacturer’s instructions. Slides were baked in a dry oven for 1 h at 60 °C to promote tissue-to-slide adherence, deparaffinized and rehydrated in fresh xylenes and 100% ethanol, and air dried. RNAscope® hydrogen peroxide (Advanced Cell Diagnostics) was next applied to each tissue section for 10 min (min) at RT to block endogenous peroxidase activity, followed by rinsing with fresh distilled water (dH_2_O). Disruption of formalin cross-linking and unmasking of antigenic epitopes was achieved by submerging slides in a boiling 1X RNAscope® target retrieval solution (Advanced Cell Diagnostics) for 15 min, followed by rinsing with fresh dH_2_O and 100% ethanol. Once slides had completely air dried, a hydrophobic barrier was drawn around each tissue using an ImmEdge™ pen (Vector Laboratories, Burlingame, CA), and slides were stored at RT overnight with desiccants. The following day, RNAscope® Protease Plus was applied to each tissue section and incubated in a humidifying tray at 40 °C in a HybEZ™ Hybridization System oven (Advanced Cell Diagnostics) for 30 min. Slides were then rinsed with fresh dH_2_O before proceeding to probe hybridization.

Probe hybridization, amplification, and detection were performed according to manufacturer’s instructions. All incubations were carried out in a humidifying tray either at RT or in a HybEZ™ oven at 40 °C. Between each incubation step, slides were washed with fresh 1X Wash Buffer (Advanced Cell Diagnostics). To allow binding of the ZZ probes to target mRNA, customized probes, prewarmed to 40 °C were applied to each tissue section and incubated at 40 °C for 2 h. Branched amplification and detection of the probes with Fast Red chromogen (Advanced Cell Diagnostics) was achieved by incubating slides with kit reagents (Advanced Cell Diagnostics) as follows: AMP 1 (30 min), AMP 2 (15 min), AMP 3 (30 min), and AMP 4 (15 min) at 40 °C; AMP 5 (30 min) and AMP 6 (15 min) at RT; and a 60:1 solution of RED-A: RED-B (Advanced Cell Diagnostics) at RT for 10 min.

Following RED detection, slides were rinsed with fresh dH_2_O before being transferred to a 1:1 Gill’s hematoxylin I:dH_2_O (American MasterTech, Lodi, CA) counterstain. Slides were submerged in hematoxylin solution for 2 min, rinsed with fresh dH_2_O thrice, submerged in 0.02% ammonia water for bluing, and dry baked at 40 °C for 20 min or until completely dry. Tissue dehydration was not completed due to the alcohol-sensitive nature of the Fast Red chromogen. To mount the tissue samples, slides were dipped in fresh xylenes, 1–2 drops of aqueous EcoMount mounting medium (Biocare Medical, Pacheco, CA) was applied to each tissue section, and a #1 thickness cover slip was applied over top of the tissue section. Slides were dried at RT in the dark overnight before microscopic examination.

## Results

### Kinetics of granuloma and follicle-like structures following *M. bovis* challenge

**Fig. 1:**
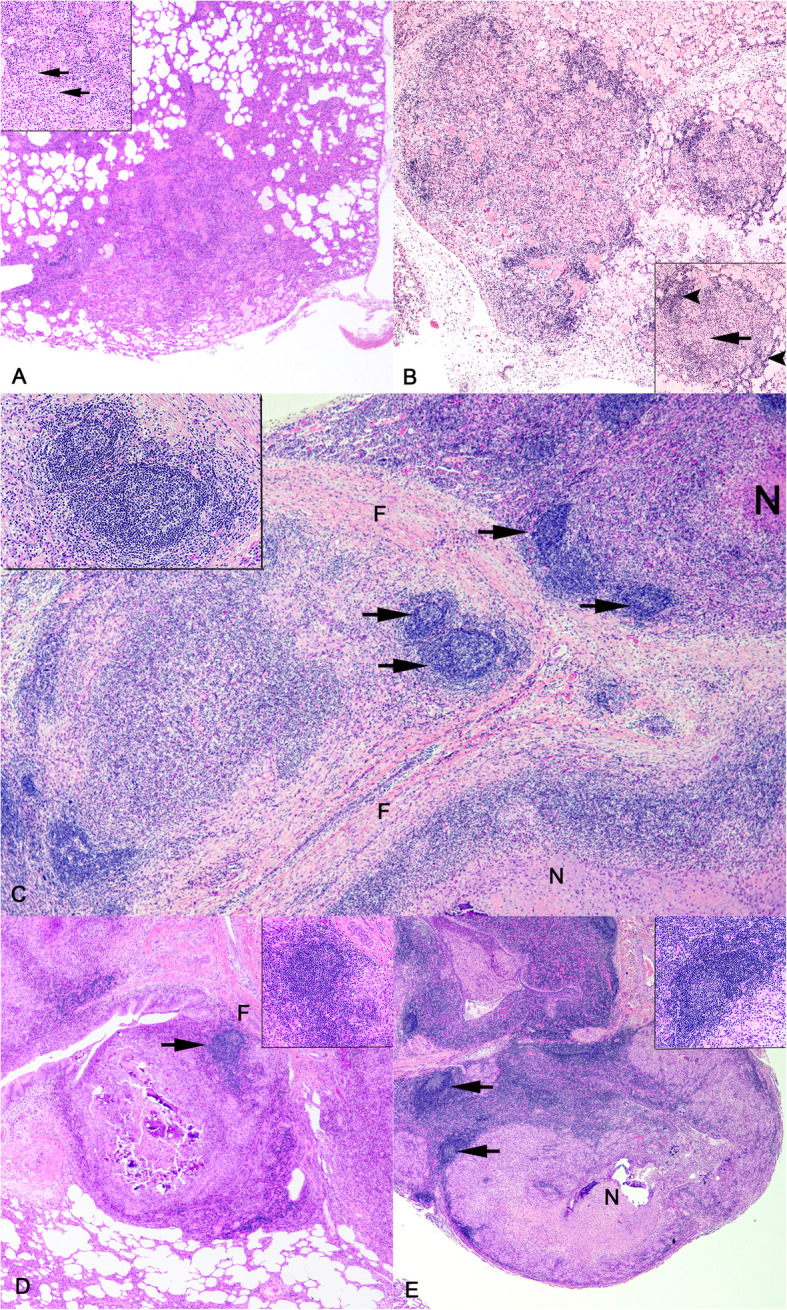
Kinetics of pulmonary granuloma formation following virulent *M. bovis* challenge. **A** Granulomas at 15 days post-infection (dpi). Inset: macrophages filling alveolar spaces (arrows). **B** Granulomas at 30 dpi. Inset: macrophages filling alveolar spaces (long arrow) and alveolar interstitium with increased infiltrates of lymphocytes peripheral to accumulations of macrophages (arrowheads). **C**-**E** Granulomas at 90 dpi, 180 dpi and 270 dpi, respectively. Granuloma-associated follicle-like structures (arrows). Insets: germinal centers surrounded by a denser zone of lymphocytes. *N* = necrotic center, F = fibrous capsule, HE. Original magnification 10X (**A**, **B**, **D**, **E**), 20X (**C**)

At various time points after *M. bovis* challenge, tissue sections of lung were collected at necropsy and evaluated grossly and microscopically for granulomas. All animals, regardless of time point presented with pulmonary granulomas, although their number, size, and stage varied as did the individual granuloma bacterial burden [5]. As described previously [23], at 15 days after infection, there were typical stage I microscopic lesions characterized microscopically as small, poorly organized infiltrates of macrophages that variably filled alveoli and increased numbers of lymphocytes in the alveolar interstitium; at times lymphocytic infiltrates surrounded blood vessels or lymphatics (Fig. 1A).

At 30 days after infection, lesions similar to those seen at 15 days were evident in addition to type 2 lesions being small, more organized granulomas composed of macrophages and loosely surrounded by scattered lymphocytes and lymphocytes expanding alveolar interstitium with no follicle-like organization (Fig. 1B).

In contrast, analysis of samples collected at 90, 180 and 270 days after infection revealed well organized stages 3, 4, and 5 necrotizing and non-necrotizing granulomas with macrophage rich zones surrounded by peripheral rims of lymphocytes present both in a scattered fashion, as well as organized into lymphoid follicle-like structures (Fig. 1C-E). Follicle-like structures were seen internal to collagenous bands of the fibrous capsule, as well as within the capsule separating collagenous fibers. These data are consistent with our previous reports of the temporal kinetics of granuloma formation in cattle, [5, 23]. No granulomas were seen in age-matched non-infected steers.

### Cellular composition of granuloma associated lymphoid follicle-like structures

**Fig. 2:**
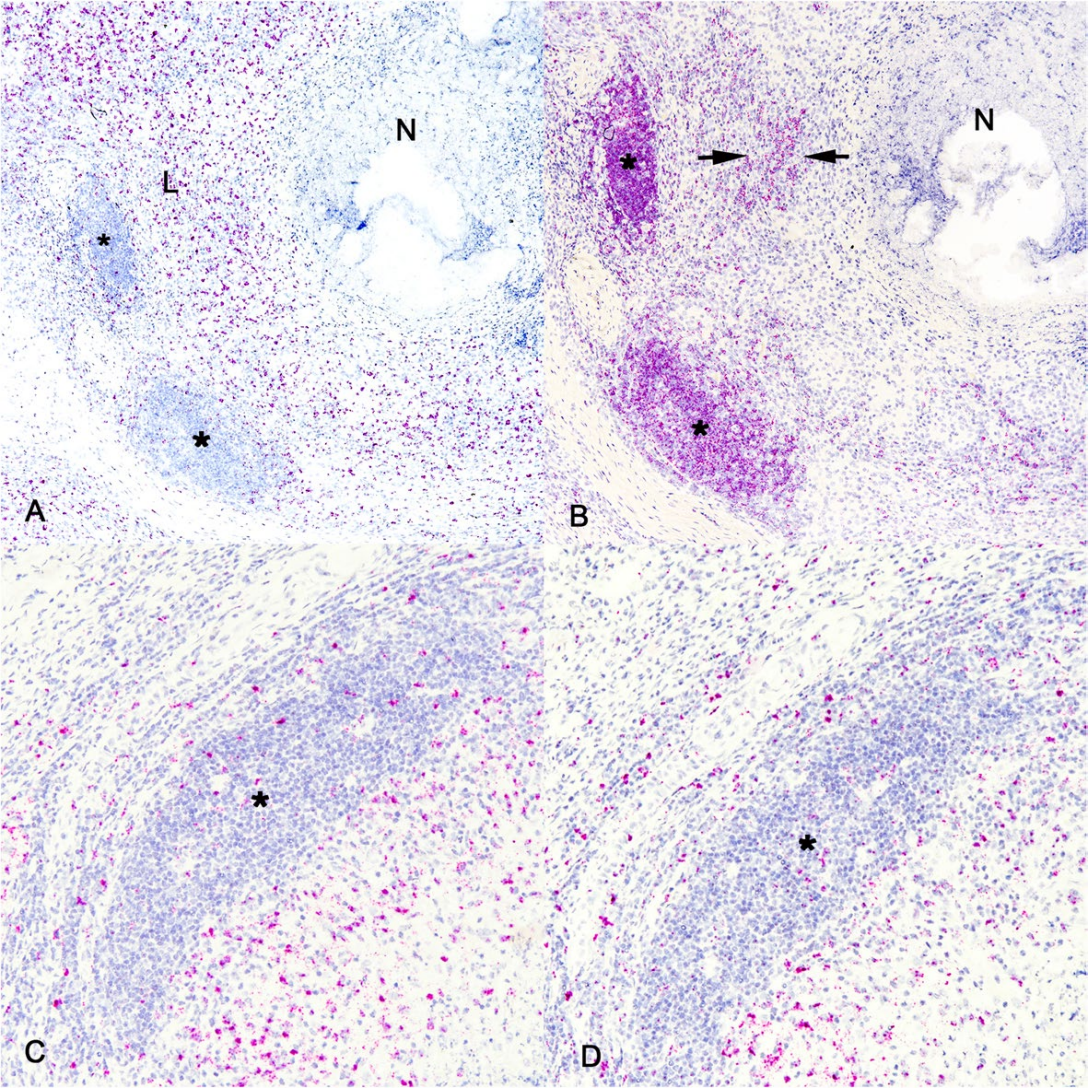
Lymphoid and myeloid composition of pulmonary granulomas and their associated follicles. Granulomas at 90 days dpi. **A** CD3ε positive ISH labeling is scattered within the lymphocyte rich zone of granuloma (L), with lesser labeling within follicle-like structures (asterisks) and no labeling in necrotic area (N). **B** CD79 positive ISH labeling of scattered B cells in lymphocyte rich zone of granuloma (between arrows) and cells arranged in follicle-like structures (asterisks). Punctate ISH labeling for CD11b (**C**) and CD11c (**D**) within and around follicle-like structure (asterisks). ISH labeling for CD3ε, CD79, CD11b and CD11c. Original magnification 100X

For this analysis samples collected 90–270 days after infection were used, since the observed follicle-like structures were present at these time points. In situ hybridization labeling of mRNA of the epsilon polypeptide of the T cell receptor (CD3ε) showed labeling of scattered individualized CD3ε mRNA positive T cells generally limited to the periphery of the granuloma, within the lymphocyte-rich zone and not within the follicle like-structures (Fig. 2A). This pattern of staining was consistent at all time points analyzed. For detection of the B cell antigen receptor (CD79) mRNA, ISH was used to determine the B cell composition within granuloma-associated follicle-like structures. In contrast to the CD3ε labeling, CD79 mRNA positive cells were most numerous within follicle-like structures but were also scattered throughout the lymphocyte-rich zone (Fig. 2B). This pattern of staining was consistent at all time points analyzed. Altogether, these data suggest that the granuloma-associated follicle-like structures are primarily composed of CD79 mRNA expressing cells (B cells) and not CD3ε mRNA expressing cells (T cells).

Tissue sections were also assessed for the presence of myeloid cells. ISH for mRNA for the integrin alpha M (*ITGAM*) gene, also known as CD11b, was used to target monocytes, while ISH for mRNA for the integrin alpha X (*ITGAX)* gene that encodes for integrin CD11c, was used to identify dendritic cells. In pulmonary granulomas, scattered labeling of CD11b (Fig. 2C) and CD11c (Fig. 2D) mRNA positive cells was seen within the macrophage-rich and lymphocyte-rich zones, around follicle-like structures, and to a lesser extent within those structures. This pattern of staining was consistent at all time points analyzed. This is consistent with the location of these cells in the paracortex, T cell rich zone of lymph nodes and not within follicles.

### Molecular characterization of granuloma-associated follicle-like structures

**Fig. 3:**
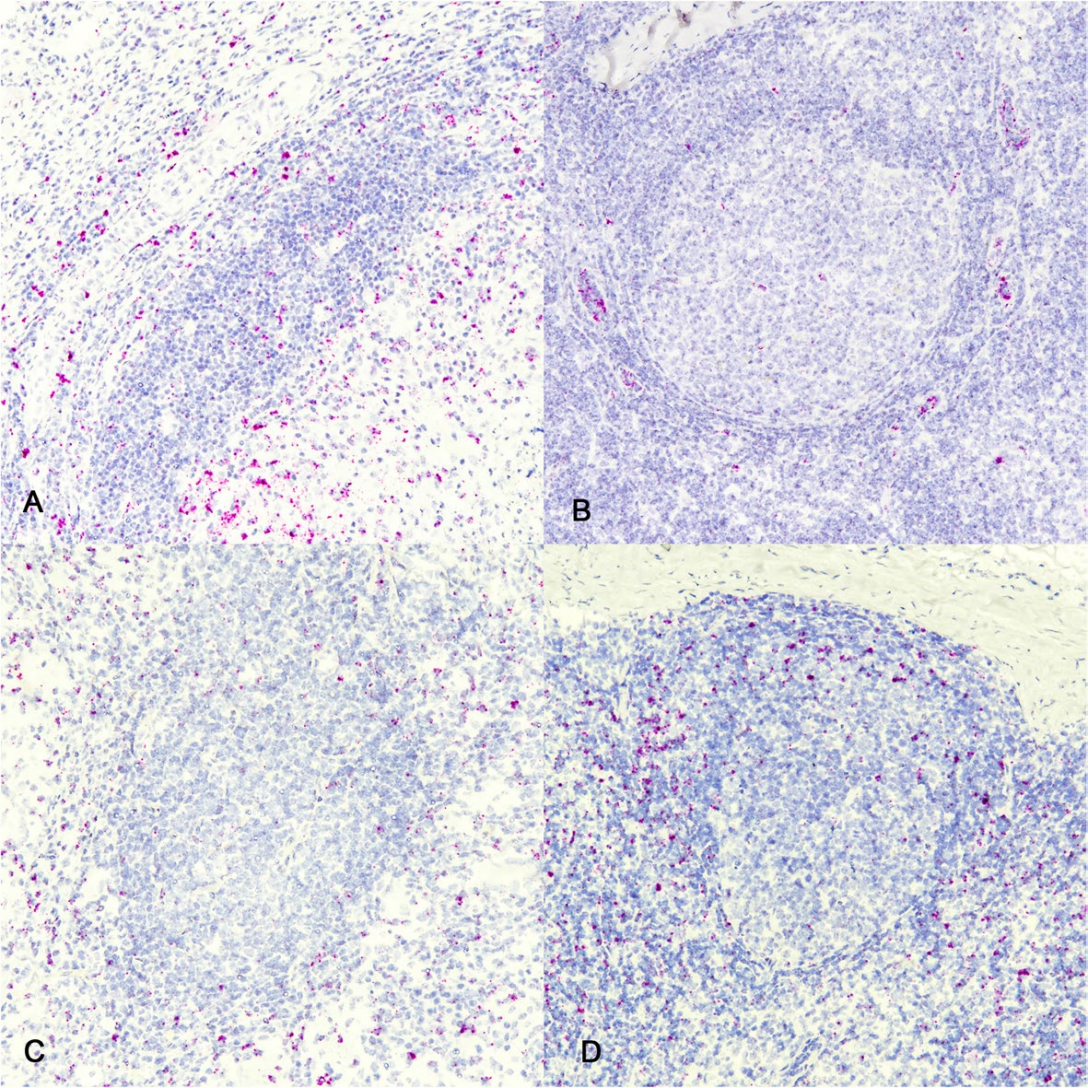
Molecular characterization of granuloma-associated TLS. Granulomas at 90 days dpi. ISH mRNA labeling for CD34 and ICOS. **A** Note punctate and linearly oriented ISH labeling for CD34 around, and to a lesser extent within granuloma associated follicle-like structures and on sections of tracheobronchial lymph node from a non-infected age matched steer (**B**). **C** Note punctate labeling around and to a lesser extent within granuloma associated follicle-like structure and around and to a lesser extent within the follicle of a section of tracheobronchial lymph node from a non-infected age-matched steer (**D**). ISH labeling for CD34 and ICOS. Original magnification 100X

Tertiary lymphoid structures are defined as lymphoid aggregates with organized fibrovascular elements such as high-endothelial venules (HEV) and lymphatic vessels. The cell adhesion molecule L-selectin, CD34 is found on endothelial cells, including HEVs. ISH labeling demonstrated CD34 mRNA positive cells surrounding granuloma-associated TLSs (Fig. 3A). This pattern of staining was consistent at all time points analyzed. As a control to ensure proper labeling with the CD34 probe sections of tracheobronchial lymph nodes from non-infected age-matched steers were also examined. The pattern of CD34 mRNA labeling in lymph nodes was similar to that seen above, showing labeling around the follicle (Fig. 3B). In both granulomas and lymph node sections the pattern of labeling was often linear, consistent with labeling of a component of the vascular system.

The inducible costimulatory (ICOS) molecule, also known as CD278, is a cell-surface protein found on activated T cells. In the context of TLSs, ICOS/ICOS-ligand interactions are critical for lymphotoxin alpha-3 (LTα3) production, which in turn promotes chemokine signals for TLS formation [45]. ICOS is expressed by T follicular helper cells (T_FH_), a distinct subset of T helper cells found within germinal centers and TLSs [46, 47]. Therefore, ICOS mRNA expression associated within granuloma associated follicle-like structures was assessed and found present in punctate fashion between, surrounding, and to a lesser extent, within the follicle-like structures (Fig. 3C). This pattern of staining was consistent at all time points analyzed. In lymph nodes, labeling was seen in the interfollicular cortex and paracortex where T cells predominate (Fig. 3D). Altogether, these data suggest that the observed granuloma-associated follicle-like structures, share the molecular signature of characterized TLSs.

### Chemokine profile of granuloma associated follicle-like structures

**Fig. 4:**
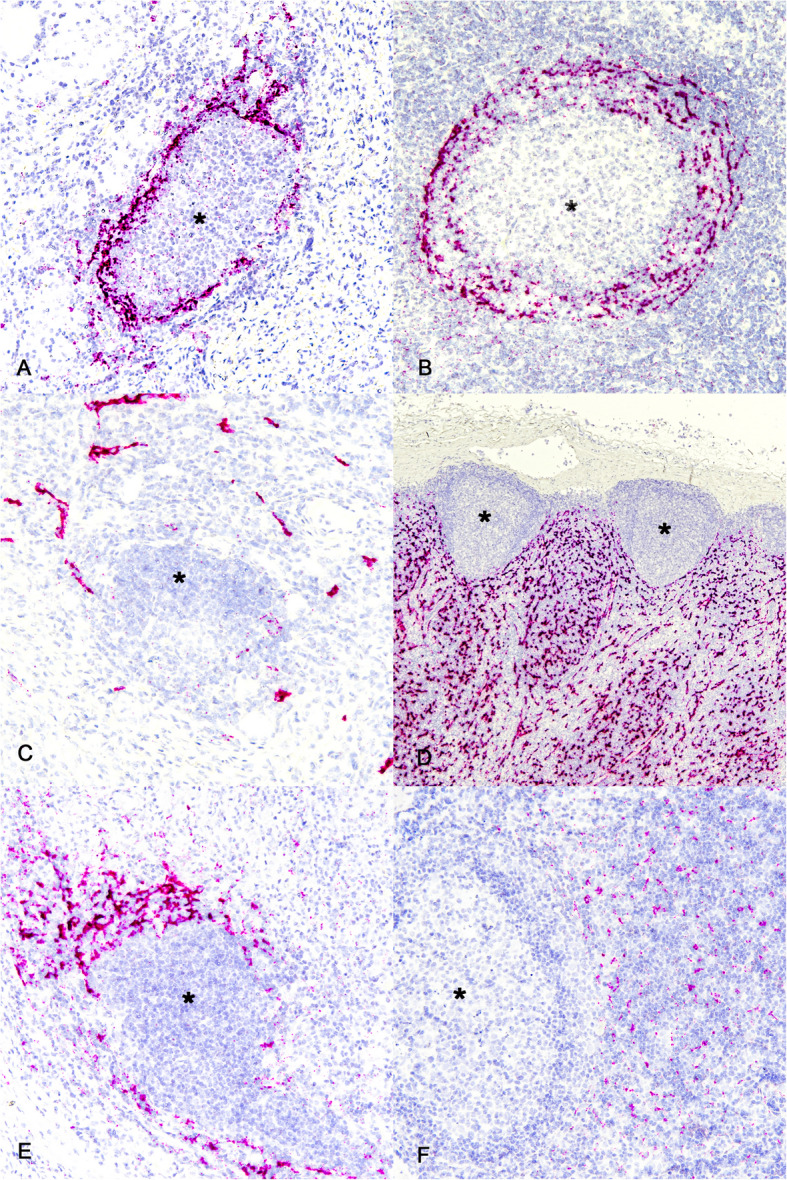
Chemokine expression profile of granuloma associated TLS. Granulomas at 90 dpi. **A** ISH labeling for CXCL13 in a granuloma associated follicle-like structure (asterisk) and (**B**) around a follicle (asterisk) from a tracheobronchial lymph node from a non-infected age-matched steer (**B**). Also, CCL21 mRNA staining outside of granuloma associated follicle-like structure (asterisk) (**C**). Staining has a linear pattern consistent with labeling of vascular or lymphatic endothelial cell labeling in lymph node sections (asterisks) (**D**). Lastly, (**E**) ISH labeling for CCL19 around follicle-like structure of granuloma with minimal labeling within follicle-like structure (asterisk). **F** Labeling is limited to the interfollicular cortex but not follicle (asterisk) of a tracheobronchial lymph node from a non-infected age-matched steer. ISH labeling for CXCL13, CCL21, CCL19. Original magnification 100X, (**D**) 20X

Chemokines play an important role in TLS formation and maintenance, specifically, CXCL13, CCL19 and CCL21 [48, 49]. CXCL13 expression is strongly correlated with the presence of TLSs in tissues, independent of CCL19 and CCL21 expression [50, 51]. Therefore, the chemokine expression profile of granuloma associated TLS from infected calves was compared to the labeling observed in lymph nodes from age matched, non-infected calves. Labeling of mRNA for the B cell selective chemokine CXCL13, also known as B-lymphocyte attractant, was markedly present at the periphery of granuloma associated follicle-like structures (Fig. 4A). This pattern of staining was consistent at all time points analyzed. Similar staining was observed surrounding follicles in lymph nodes (Fig. 4B).

CCL21 is constitutively expressed in SLOs by stromal cells and endothelial cells including high endothelial venules and mediates CCR7 + naïve and central memory T cell extravasation [52]. In non-lymphoid tissues overexpression of CCL21 is sufficient to activate pathways that lead to B and T cell recruitment in ectopic sites such as TLSs [49, 52]. Labeling of CCL21 mRNA was seen surrounding granuloma associated follicle-like structures and had a noticeable linear appearance consistent with expression on lymphatic or vascular endothelial cells (Fig. 4C). This pattern of staining was consistent at all time points analyzed. Similarly, expression of CCL21 mRNA in lymph nodes was abundant in the interfollicular cortex, paracortex and medulla, but not within follicles, and often seen in a linear pattern consistent with endothelial cell labeling (Fig. 4D).

CCL19 is expressed by stromal cells in the lymph node paracortex T cell zone and binds to the CCR7 receptor on T and B cells. In the granuloma associated follicle-like structures ISH labeling was primarily limited to the area around the follicle-like structures (Fig. 4E). This pattern of staining was consistent at all time points analyzed. Similarly, CCL19 mRNA labeling in lymph nodes was present in the interfollicular cortex and paracortex outside of lymphoid follicles but was noticeably less abundant and less intense than that associated with granuloma associated follicle-like structures (Fig. 4F).

## Discussion

Altogether, the data presented here demonstrate that bovine pulmonary tuberculous granulomas develop follicle-like structures with compartmentalized T and B cell areas, myeloid cells, vascular elements, and cytokine expression consistent with that described for TLSs observed in humans, mice and non-human primates [30, 34]. Moreover, granuloma associated TLSs described here share many similarities with SLOs, specifically, bovine lymph nodes.

Immune responses are generally aimed at pathogen control. Some pathogens, such as *M. bovis* are not eliminated due to the physical properties of the organism, as well as pathogen driven mechanisms that counter host responses. The result is a sustained immune response that results in chronic inflammation, that can maintain premunition but can also lead to increasing tissue destruction and progressing clinical signs. These two outcomes are on spectrum of protection vs disease and the final endpoint is dependent upon multiple host and pathogen specific factors. Chronic inflammatory reactions such as the tuberculous granuloma are infiltrated by effector cells of the immune system such as T cells, macrophages, and multinucleated giant cells, but also B cells.

Emerging evidence suggests a role for immunoglobulins and B cells in controlling obligate and facultative intracellular pathogens such as *Leishmania* spp., *Chlamydia* spp., and *Salmonella* spp. [53–55]. *Mycobacteria* spp. are obligate intracellular pathogens for which cell-mediated immunity and CD4 + T cells are considered critical for protection. However, B cells have also been described in tuberculous granulomas from humans, mice, non-human primates, goats, and cattle [30, 37, 56–60].

Within the granuloma, cellular elements (T cells, B cells, macrophages) organize themselves into distinct compartments. B cells may be present as scattered infiltrates or organized into anatomically recognizable lymphoid follicles known as TLSs. Tertiary lymphoid structures have many characteristics of lymphoid follicles in SLOs such as lymph nodes. Lymphoid neogenesis is the result of coordinated interaction between lymphoid and non-lymphoid cells, adhesion molecules, chemokines, and cytokines. It is a dynamic process where unorganized infiltrates of lymphocytes aggregate, forming B cell follicles and germinal centers [49]. Chronic inflammatory lesions that are highly infiltrated with lymphocytes such as tuberculous granulomas tend to have the most organized TLSs [49].

In the present study lymphoid follicle-like structures in bovine pulmonary tuberculous granulomas were shown to resemble follicles of SLOs and are consistent with TLSs. Functionally, both are associated with adaptive immune responses and can contain germinal centers where cognate T cell and B cell interactions occur [61].

TLSs resemble SLOs in their cellular composition (T cells, B cells, antigen presenting cells), germinal centers, vascular and lymphatic conduits. Compartmentalization of T and B cell zones is due in part to the presence of the T cell cytokines CCL19 and CCL21, and the B cell chemokine CXCL13. In particular, CXCL13 gene expression is highly correlative with TLS presence, unlike CCL19 and CCL21, which have more nuanced effects on TLS maturity and spatial organization [50, 51]. The well-defined capsule of lymph nodes with a subcapsular sinus is not present in TLSs. The presence of organized B cell follicles in close proximity to tuberculous granulomas prompts the question about the role these cells play in host response to tuberculous mycobacteria. However, studies to examine the function of B cells and TLSs in mycobacterial infections is unclear and somewhat contradictory. In one study using B cell deficient mice, it was noted that compared to wild type mice there was diminished pathology, a decreased T_H_1 response, and an increased level of IL-10. It was suggested that B cells and TLSs contribute to the development of exacerbated pathology, based in part on the their presence in granulomas manifesting necrosis and cavitation, as well as the ability of B cells to restrict expression of the anti-inflammatory and immunosuppressive effects of IL-10 [57]. However, in a separate study using a different strain of B cell deficient mice it was concluded that B cells play a protective and anti-inflammatory role in control of *M. tb* infection [58].

In a macaque model of human TB, B cell depletion resulted in altered cytokine and T cell responses, with higher bacterial burden and lower levels of inflammation compared to B cell replete animals [62]. It has been suggested that TLSs represent a more efficient way of presenting antigen and driving the immune response at lesion sites [32]. In the macaque model an increased amount of antigen-specific IgG at the site of infection is believed to modulate host–pathogen interactions within the granuloma [32]. In tuberculous granulomas from humans, granuloma associated TLSs are composed of B cells and mycobacteria containing antigen presenting cells surrounded by CD4 + and CD8 + T cells. It is suggested that these TLSs become a major area of host–pathogen crosstalk [30].

Host–pathogen interactions within TLSs have been observed in other chronic conditions, such as *Brucella abortus* infection where myeloid cells in omental fat associated TLSs exhibited immunomodulatory and immunosuppressive effects in mice and humans [63]. Studies of Influenza A and SARS-CoV-2 have demonstrated that TLSs, specifically inducible bronchus associated lymphoid tissue (iBALT), are critical in viral clearance and vaccine induced immune responses by facilitating B-cell selection and maturation [64, 65]. These findings highlight the need for a deeper understanding of the functions of TLSs in both human and veterinary medicine.

In the data presented here, the emergence of pulmonary granuloma associated follicle-like structures following *M. bovis* infection was described by light microscopy and ISH. The appearance of these structure is time dependent, and their structure and molecular signatures match those of lymph node follicles and resemble previously characterized TLSs. The function of these structures in the bovine tuberculous granuloma is not fully understood. Given that clinical disease in cattle may take years to develop, and clearance of *M. bovis* has not been documented, one hypothesis may be that the presence of these TLSs is detrimental. However, one could also argue that the alternative hypothesis is plausible, in that the presence of TLSs allows the animal to control disease for an extended period and establishes a state of premunition which has been shown to be beneficial in macaques rechallenged with *M. tuberculosis* [24]. Further assessment of these structures, as they relate to protection upon rechallenge and as they relate to animals that do become clinical, may shed some light on their function.

In this study, the function of B cells within the TLSs examined was not investigated. Additionally, the current analysis was limited to qualitative evaluation of ISH labeling at various time points and no quantitative measures were performed to determine changes in mRNA expression over time, although TLSs were not observed until 90 days after infection. The current study focused on granulomas of the lung as examination of TLSs in other species have focused on pulmonary granulomas as well.

## Data Availability

The original contributions presented in the study are included in the article/supplementary material, further inquiries can be directed to the corresponding author(s).

## References

[1] Armstrong DT, Eisemann E, Parrish N. A brief update on Mycobacterial taxonomy, 2020–2022. J Clin Microbiol. 2023;61(4):e00331-e322. 36951562 10.1128/jcm.00331-22 PMC10117096

[2] Lin PL, Coleman T, Carney JP, Lopresti BJ, Tomko J, Fillmore D, et al. Radiologic responses in cynomolgous macaques for assessing tuberculosis chemotherapy regimens. Antimicrob Agents Chemother. 2013;57(9):4237–44. 23796926 10.1128/AAC.00277-13 PMC3754323

[3] Lin PL, Ford CB, Coleman MT, Myers AJ, Gawande R, Ioerger T, et al. Sterilization of granulomas is common in active and latent tuberculosis despite within-host variability in bacterial killing. Nat Med. 2014;20(1):75–9. 24336248 10.1038/nm.3412 PMC3947310

[4] Gideon HP, Skinner JA, Baldwin N, Flynn JL, Lin PL. Early whole blood transcriptional signatures are associated with severity of lung inflammation in cynomolgus macaques with *Mycobacterium tuberculosis* infection. J Immunol. 2016;197(12):4817–28. 27837110 10.4049/jimmunol.1601138 PMC5289749

[5] Palmer MV, Thacker TC, Kanipe C, Boggiatto PM. Heterogeneity among pulmonary granulomas in cattle experimentally infected with *Mycobacterium bovis*. Front Vet Sci. 2021;8:671460. 34026898 10.3389/fvets.2021.671460 PMC8138452

[6] Palmer MV, Thacker TC, Waters WR. Analysis of cytokine gene expression using a novel chromogenic in-situ hybridization method in pulmonary granulomas of cattle infected experimentally by aerosolized *Mycobacterium bovis*. J Comp Pathol. 2015;153(2–3):150–9. 26189773 10.1016/j.jcpa.2015.06.004

[7] Palmer MV, Thacker TC, Waters WR. Differential cytokine gene expression in granulomas from lungs and lymph nodes of cattle experimentally infected with aerosolized *Mycobacterium bovis*. PLoS One. 2016;11(11):e0167471. 27902779 10.1371/journal.pone.0167471 PMC5130274

[8] Palmer MV, Waters WR, Thacker TC. Lesion development and immunohistochemical changes in granulomas from cattle experimentally infected with *Mycobacterium bovis*. Vet Pathol. 2007;44(6):863–74. 18039899 10.1354/vp.44-6-863

[9] Shu D, Heiser A, Wedlock DN, Luo D, de Lisle GW, Buddle BM. Comparison of gene expression of immune mediators in lung and pulmonary lymph node granulomas from cattle experimentally infected with *Mycobacterium bovis*. Vet Immunol Immunopathol. 2014;160(1–2):81–9. 24852075 10.1016/j.vetimm.2014.03.017

[10] Johnson L, Gough J, Spencer Y, Hewinson G, Vordermeier M, Wangoo A. Immunohistochemical markers augment evaluation of vaccine efficacy and disease severity in bacillus Calmette-Guerin (BCG) vaccinated cattle challenged with *Mycobacterium bovis*. Vet Immunol Immunopathol. 2006;111(3–4):219–29. 16540176 10.1016/j.vetimm.2006.01.016

[11] Wangoo A, Johnson L, Gough J, Ackbar R, Inglut S, Hicks D, et al. Advanced granulomatous lesions in *Mycobacterium bovis*-infected cattle are associated with increased expression of type I procollagen, gamma delta (WC1+) T cells and CD 68+ cells. J Comp Pathol. 2005;133(4):223–34. 16154140 10.1016/j.jcpa.2005.05.001

[12] Aranday-Cortes E, Bull NC, Villarreal-Ramos B, Gough J, Hicks D, Ortiz-Pelaez A, et al. Upregulation of IL-17A, CXCL9 and CXCL10 in early-stage granulomas induced by *Mycobacterium bovis* in cattle. Transbound Emerg Dis. 2013;60(6):525–37. 22909117 10.1111/j.1865-1682.2012.01370.x

[13] Salguero FJ, Gibson S, Garcia-Jimenez W, Gough J, Strickland TS, Vordermeier HM, et al. Differential cell composition and cytokine expression within lymph node granulomas from BCG-vaccinated and non-vaccinated cattle experimentally infected with *Mycobacterium bovis*. Transbound Emerg Dis. 2017;64(6):1734–49. 27615603 10.1111/tbed.12561

[14] Cronan MR, Beerman RW, Rosenberg AF, Saelens JW, Johnson MG, Oehlers SH, et al. Macrophage epithelial reprogramming underlies mycobacterial granuloma formation and promotes infection. Immunity. 2016;45(4):861–76. 27760340 10.1016/j.immuni.2016.09.014 PMC5268069

[15] Cronan MR. In the thick of It: Formation of the tuberculous granuloma and its effects on host and therapeutic responses. Front Immunol. 2022;13:820134. 35320930 10.3389/fimmu.2022.820134 PMC8934850

[16] Pitzalis C, Jones GW, Bombardieri M, Jones SA. Ectopic lymphoid-like structures in infection, cancer and autoimmunity. Nat Rev Immunol. 2014;14(7):447–62. 24948366 10.1038/nri3700

[17] Jones GW, Hill DG, Jones SA. Understanding immune cells in tertiary lymphoid organ development: it is all starting to come together. Front Immunol. 2016;7:401. 27752256 10.3389/fimmu.2016.00401 PMC5046062

[18] Sato Y, Silina K, van den Broek M, Hirahara K, Yanagita M. The roles of tertiary lymphoid structures in chronic diseases. Nat Rev Nephrol. 2023;19(8):525–37. 37046081 10.1038/s41581-023-00706-z PMC10092939

[19] Schumacher TN, Thommen DS. Tertiary lymphoid structures in cancer. Science. 2022;375(6576):eabf9419. 34990248 10.1126/science.abf9419

[20] Tsai MC, Chakravarty S, Zhu G, Xu J, Tanaka K, Koch C, et al. Characterization of the tuberculous granuloma in murine and human lungs: cellular composition and relative tissue oxygen tension. Cell Microbiol. 2006;8(2):218–32. 16441433 10.1111/j.1462-5822.2005.00612.x

[21] Wedlock DN, Denis M, Vordermeier HM, Hewinson RG, Buddle BM. Vaccination of cattle with Danish and Pasteur strains of *Mycobacterium bovis* BCG induce different levels of IFN gamma post-vaccination, but induce similar levels of protection against bovine tuberculosis. Vet Immunol Immunopathol. 2007;118(1–2):50–8. 17524495 10.1016/j.vetimm.2007.04.005

[22] Waters WR, Palmer MV, Buddle B, Vordermeier HM. Bovine tuberculosis vaccine research: Historical perspectives and recent advances. Vaccine. 2012;30(16):2611–22. 22342705 10.1016/j.vaccine.2012.02.018

[23] Palmer MV, Wiarda J, Kanipe C, Thacker TC. Early pulmonary lesions in cattle infected via aerosolized *Mycobacterium bovis*. Vet Pathol. 2019;56:544. 30895908 10.1177/0300985819833454

[24] Cadena AM, Hopkins FF, Maiello P, Carey AF, Wong EA, Martin CJ, et al. Concurrent infection with *Mycobacterium tuberculosis* confers robust protection against secondary infection in macaques. PLoS Pathog. 2018;14(10):e1007305. 30312351 10.1371/journal.ppat.1007305 PMC6200282

[25] Peters NC, Pagan AJ, Lawyer PG, Hand TW, Henrique Roma E, Stamper LW, et al. Chronic parasitic infection maintains high frequencies of short-lived Ly6C+CD4+ effector T cells that are required for protection against re-infection. PLoS Pathog. 2014;10(12):e1004538. 25473946 10.1371/journal.ppat.1004538 PMC4256462

[26] Belkaid Y, Piccirillo CA, Mendez S, Shevach EM, Sacks DL. CD4+CD25+ regulatory T cells control *Leishmania major* persistence and immunity. Nature. 2002;420(6915):502–7. 12466842 10.1038/nature01152

[27] Sergent E, Parrot L, Donatien A. On the necessity of having a term to express the reistance of carrieres of germs to superimposed infections. Trans R Soc Trop Med Hyg. 1925;18(7):383–5.

[28] Rijnink WF, Ottenhoff THM, Joosten SA. B-cells and antibodies as contributors to effector immune responses in tuberculosis. Front Immunol. 2021;12:640168. 33679802 10.3389/fimmu.2021.640168 PMC7930078

[29] Chan J, Mehta S, Bharrhan S, Chen Y, Achkar JM, Casadevall A, et al. The role of B cells and humoral immunity in *Mycobacterium tuberculosis* infection. Semin Immunol. 2014;26(6):588–600. 25458990 10.1016/j.smim.2014.10.005 PMC4314354

[30] Ulrichs T, Kosmiadi GA, Trusov V, Jorg S, Pradl L, Titukhina M, et al. Human tuberculous granulomas induce peripheral lymphoid follicle-like structures to orchestrate local host defence in the lung. J Pathol. 2004;204(2):217–28. 15376257 10.1002/path.1628

[31] Kahnert A, Hopken UE, Stein M, Bandermann S, Lipp MA, Kaufmann SHE. *Mycobacterium tuberculosis* triggers formation of lymphoid structure in murine lungs. J Infect Dis. 2007;195:46–54. 17152008 10.1086/508894

[32] Phuah JY, Mattila JT, Lin PL, Flynn JL. Activated B cells in the granulomas of nonhuman primates infected with *Mycobacterium tuberculosis*. Am J Pathol. 2012;181(2):508–14. 22721647 10.1016/j.ajpath.2012.05.009 PMC3409439

[33] Fuller CL, Flynn JL, Reinhart TA. In situ study of abundant expression of proinflammatory chemokines and cytokines in pulmonary granulomas that develop in cynomolgus macaques experimentally infected with *Mycobacterium tuberculosis*. Infect Immun. 2003;71(12):7023–34. 14638792 10.1128/IAI.71.12.7023-7034.2003 PMC308896

[34] Pipi E, Nayar S, Gardner DH, Colafrancesco S, Smith C, Barone F. Tertiary lymphoid structures: autoimmunity goes local. Front Immunol. 1952;2018:9. 10.3389/fimmu.2018.01952 PMC6143705 30258435

[35] Ruddle NH. High endothelial venules and lymphatic vessels in tertiary lymphoid organs: characteristics, functions, and regulation. Front Immunol. 2016;7:491. 27881983 10.3389/fimmu.2016.00491 PMC5101196

[36] Yang F, Yang J, Wu M, Chen C, Chu X. Tertiary lymphoid structures: new immunotherapy biomarker. Front Immunol. 2024;15:1394505. 39026662 10.3389/fimmu.2024.1394505 PMC11254617

[37] Cassidy JP, Bryson DG, Gutierrez Cancela MM, Forster F, Pollock JM, Neill SD. Lymphocyte subtypes in experimentally induced early-stage bovine tuberculous lesions. J Comp Pathol. 2001;124(1):46–51. 11428188 10.1053/jcpa.2000.0427

[38] Waters WR, Thacker TC, Nelson JT, DiCarlo DM, Maggioli MF, Greenwald R, et al. Virulence of two strains of *Mycobacterium bovis* in cattle following aerosol infection. J Comp Pathol. 2014;151(4):410–9. 25306158 10.1016/j.jcpa.2014.08.007

[39] Larsen MH, Biermann K, Jacobs WR Jr. Laboratory maintenance of *Mycobacterium tuberculosis*. Curr Protoc Microbiol. 2007;Chapter 10:Unit 10A 11. 10.1002/9780471729259.mc10a01s6 18770602

[40] Palmer MV, Waters WR, Whipple DL. Aerosol delivery of virulent *Mycobacterium bovis* to cattle. Tuberculosis (Edinb). 2002;82(6):275–82. 12623270 10.1054/tube.2002.0341

[41] Garber JC. Guide for the care and use of laboratory animals. 8th ed. Washington, D.C.: The National Academies Press; 2011. 21595115

[42] FASS. Guide for the care and use of agricultural animals in research and teaching, Third edn. Champaign, IL: Federation of Animal Science Societies; 2010.

[43] Rayner EL, Pearson GR, Hall GA, Basaraba RJ, Gleeson F, McIntyre A, et al. Early lesions following aerosol infection of rhesus macaques (*Macaca mulatta*) with *Mycobacterium tuberculosis* strain H37RV. J Comp Pathol. 2013;149(4):475–85. 23880551 10.1016/j.jcpa.2013.05.005

[44] Wang F, Flanagan J, Su N, Wang LC, Bui S, Nielson A, et al. RNAscope^Ⓡ^: a novel in situ RNA analysis platform for formalin-fixed, paraffin-embedded tissues. J Mol Diagn. 2012;14(1):22–9. 22166544 10.1016/j.jmoldx.2011.08.002 PMC3338343

[45] Nayar S, Pontarini E, Campos J, Berardicurti O, Smith CG, Asam S, et al. Immunofibroblasts regulate LTalpha3 expression in tertiary lymphoid structures in a pathway dependent on ICOS/ICOSL interaction. Commun Biol. 2022;5(1):413. 35508704 10.1038/s42003-022-03344-6 PMC9068764

[46] Xie MM, Dent AL. Unexpected help: Follicular regulatory T cells in the germinal center. Front Immunol. 2018;9:1536. 30013575 10.3389/fimmu.2018.01536 PMC6036241

[47] Tertiary lymphoid structures methods and protocols. Hatfield, Hertfordshire. UK: Humana Press; 2018.

[48] Barone F, Bombardieri M, Manzo A, Blades MC, Morgan PR, Challacombe SJ, et al. Association of CXCL13 and CCL21 expression with the progressive organization of lymphoid-like structures in Sjogren’s syndrome. Arthritis Rheum. 2005;52(6):1773–84. 15934082 10.1002/art.21062

[49] Aloisi F, Pujol-Borrell R. Lymphoid neogenesis in chronic inflammatory diseases. Nat Rev Immunol. 2006;6(3):205–17. 16498451 10.1038/nri1786

[50] Ukita M, Hamanishi J, Yoshitomi H, Yamanoi K, Takamatsu S, Ueda A, et al. CXCL13-producing CD4+ T cells accumulate in the early phase of tertiary lymphoid structures in ovarian cancer. JCI Insight. 2022;7(12):e157215. 35552285 10.1172/jci.insight.157215 PMC9309049

[51] Groeneveld CS, Fontugne J, Cabel L, Bernard-Pierrot I, Radvanyi F, Allory Y, et al. Tertiary lymphoid structures marker CXCL13 is associated with better survival for patients with advanced-stage bladder cancer treated with immunotherapy. Eur J Cancer. 2021;148:181–9. 33743486 10.1016/j.ejca.2021.01.036

[52] Manzo A, Bugatti S, Caporali R, Prevo R, Jackson DG, Uguccioni M, et al. CCL21 expression pattern of human secondary lymphoid organ stroma is conserved in inflammatory lesions with lymphoid neogenesis. Am J Pathol. 2007;171(5):1549–62. 17982129 10.2353/ajpath.2007.061275 PMC2043516

[53] Yang X, Brunham RC. Gene knockout B cell-deficient mice demonstrate that B cells play an important role in the initiation of T cell responses to *Chlamydia trachomatis* (mouse pneumonitis) lung infection. J Immunol. 1998;161(3):1439–46. 9686609

[54] Nanton MR, Way SS, Shlomchik MJ, McSorley SJ. Cutting edge: B cells are essential for protective immunity against Salmonella independent of antibody secretion. J Immunol. 2012;189(12):5503–7. 23150714 10.4049/jimmunol.1201413 PMC3518619

[55] Gibson-Corley KN, Boggiatto PM, Bockenstedt MM, Petersen CA, Waldschmidt TJ, Jones DE. Promotion of a functional B cell germinal center response after Leishmania species co-infection is associated with lesion resolution. Am J Pathol. 2012;180(5):2009–17. 22429963 10.1016/j.ajpath.2012.01.012 PMC3349825

[56] Ulrichs T, Kosmiadi GA, Jorg S, Pradl L, Titukhina M, Mishenko V, et al. Differential organization of the local immune response in patients with active cavitary tuberculosis or with nonprogressive tuberculoma. J Infect Dis. 2005;192:89–97. 15942898 10.1086/430621

[57] Chen Y, Bharrhan S, Xu J, Sharma T, Wang Y, Salgame P, et al. B cells promote granulomatous inflammation during chronic *Mycobacterium tuberculosis* infection in mice. PLoS Pathog. 2023;19(3):e1011187. 36888692 10.1371/journal.ppat.1011187 PMC9994760

[58] Choreno-Parra JA, Bobba S, Rangel-Moreno J, Ahmed M, Mehra S, Rosa B, et al. *Mycobacterium tuberculosis* HN878 infection induces human-like B-cell follicles in mice. J Infect Dis. 2020;221(10):1636–46. 31832640 10.1093/infdis/jiz663 PMC7184917

[59] Choreno Parra JA, Martinez Zuniga N, Jimenez Zamudio LA, Jimenez Alvarez LA, Salinas Lara C, Zuniga J. Memory of natural killer cells: a new chance against *Mycobacterium tuberculosis*. Front Immunol. 2017;8:967. 28855906 10.3389/fimmu.2017.00967 PMC5558047

[60] Schinkothe J, Kohler H, Liebler-Tenorio EM. Characterization of tuberculous granulomas in different stages of progression and associated tertiary lymphoid tissue in goats experimentally infected with *Mycobacterium avium* subsp. *hominissuis*. Comp Immunol Microbiol Infect Dis. 2016;47:41–51. 27477506 10.1016/j.cimid.2016.05.006

[61] Swanson RV, Gupta A, Foreman TW, Lu L, Choreno-Parra JA, Mbandi SK, et al. Antigen-specific B cells direct T follicular-like helper cells into lymphoid follicles to mediate *Mycobacterium tuberculosis* control. Nat Immunol. 2023;24:855–68. 37012543 10.1038/s41590-023-01476-3 PMC11133959

[62] Phuah J, Wong EA, Gideon HP, Maiello P, Coleman MT, Hendricks MR, et al. Effects of B cell depletion on early *Mycobacterium tuberculosis* infection in cynomolgus macaques. Infect Immun. 2016;84(5):1301–11. 26883591 10.1128/IAI.00083-16 PMC4862708

[63] Pellegrini JM, Gonzalez-Espinoza G, Shayan RR, Hysenaj L, Rouma T, Arce-Gorvel V, et al. Brucella abortus impairs T lymphocyte responsiveness by mobilizing IL-1RA-secreting omental neutrophils. Nat Commun. 2025;16(1):862. 39833171 10.1038/s41467-024-55799-2 PMC11747348

[64] Tan HX, Esterbauer R, Vanderven HA, Juno JA, Kent SJ, Wheatley AK. Inducible bronchus-associated lymphoid tissues (iBALT) serve as sites of B cell selection and maturation following influenza infection in mice. Front Immunol. 2019;10:611. 30984186 10.3389/fimmu.2019.00611 PMC6450362

[65] Pilapitiya D, Lee WS, Vu MN, Kelly A, Webster RH, Koutsakos M, et al. Mucosal vaccination against SARS-CoV-2 using recombinant influenza viruses delivering self-assembling nanoparticles. Vaccine. 2025;46:126668. 39740385 10.1016/j.vaccine.2024.126668

